# Relative Contribution of Pharmacokinetics and Immune Signatures to Clinical Outcomes in Patients With HIV-associated Cryptococcal Meningitis

**DOI:** 10.1093/ofid/ofaf190

**Published:** 2025-04-02

**Authors:** Katharine E Stott, Dumizulu Tembo, Cheusisime Kajanga, Ajisa Ahmadu, Dumisan Namakhwa, Ruwanthi Kolamunnage-Dona, Chandni Sarker, Melanie Moyo, Ebbie Gondwe, Wezi Chimang’anga, Madalitso Chasweka, Reya V Shah, David S Lawrence, Thomas S Harrison, Joseph N Jarvis, David G Lalloo, William Hope, Henry C Mwandumba, The AMBITION Study Group, T Chimphambano, E Dziwani, A Kadzilimbile, S Kateta, E Kossam, C Kukacha, B Lipenga, J Ndaferankhande, M Ndalama, R Shah, A Singini, A Zambasa, J Goodall, K Lechiile, N Mawoko, T Mbangiwa, J Milburn, R Mmipi, C Muthoga, P Ponatshego, I Rulaganyang, K Seatla, N Tlhako, K Tsholo, A Bekiswa, L Boloko, H Bookholane, T Crede, L Davids, R Goliath, S Hlungulu, R Hoffman, H Kyepa, N Masina, D Maughan, T Mnguni, S Moosa, T Morar, M Mpalali, J Naude, I Oliphant, S Sayed, L Sebesho, M Shey, L Swanepoel, T Banda, T Chikaonda, G Chitulo, L Chiwoko, N Chome, M Gwin, T Kachitosi, B Kamanga, M Kazembe, E Kumwenda, M Kumwenda, C Maya, W Mhango, C Mphande, L Msumba, T Munthali, D Ngoma, S Nicholas, L Simwinga, A Stambuli, G Tegha, J Zambezi, C Ahimbisibwe, A Akampurira, A Alice, F Cresswell, J Gakuru, D Kiiza, J Kisembo, R Kwizera, F Kugonza, E Laker, T Luggya, A Lule, A Musubire, R Muyise, O Namujju, J Ndyetukira, L Nsangi, M Okirwoth, A Sadiq, K Tadeo, A Tukundane, D Williams, L Atwine, P Buzaare, M Collins, N Emily, C Inyakuwa, S Kariisa, J Mwesigye, S Niwamanya, A Rodgers, J Rukundo, I Rwomushana, M Ssemusu, G Stead, K Boyd, S Gondo, P Kufa, E Makaha, C Moyo, T Mtisi, S Mudzingwa, T Mwarumba, T Zinyandu, A Alanio, F Dromer, A Sturny-Leclere, P Griffin, S Hafeez

**Affiliations:** Antimicrobial Pharmacodynamics and Therapeutics group, Department of Pharmacology and Therapeutics, University of Liverpool, Liverpool, UK; Mucosal and Vascular Immunology Group, Malawi Liverpool Wellcome Programme, Blantyre, Malawi; Mucosal and Vascular Immunology Group, Malawi Liverpool Wellcome Programme, Blantyre, Malawi; Mucosal and Vascular Immunology Group, Malawi Liverpool Wellcome Programme, Blantyre, Malawi; Mucosal and Vascular Immunology Group, Malawi Liverpool Wellcome Programme, Blantyre, Malawi; Mucosal and Vascular Immunology Group, Malawi Liverpool Wellcome Programme, Blantyre, Malawi; Department of Health Data Science, University of Liverpool, Liverpool, UK; Department of Health Data Science, University of Liverpool, Liverpool, UK; Mucosal and Vascular Immunology Group, Malawi Liverpool Wellcome Programme, Blantyre, Malawi; Department of Medicine, Kamuzu University of Health Sciences, Blantyre, Malawi; Mucosal and Vascular Immunology Group, Malawi Liverpool Wellcome Programme, Blantyre, Malawi; Mucosal and Vascular Immunology Group, Malawi Liverpool Wellcome Programme, Blantyre, Malawi; Mucosal and Vascular Immunology Group, Malawi Liverpool Wellcome Programme, Blantyre, Malawi; Institute of Infection and Immunity, City St George's University London, London, UK; Department of Clinical Research, Faculty of Infectious and Tropical Diseases, London School of Tropical Medicine, London, UK; Botswana Harvard Health Partnership, Gaborone, Botswana; School of Pathology, Faculty of Health Sciences, University of the Witwatersrand, Johannesburg, South Africa; Institute of Infection and Immunity, City St George's University London, London, UK; MRC Centre for Medical Mycology, University Exeter, Exeter, UK; Department of Clinical Research, Faculty of Infectious and Tropical Diseases, London School of Tropical Medicine, London, UK; Botswana Harvard Health Partnership, Gaborone, Botswana; Liverpool School of Tropical Medicine, Liverpool, UK; Antimicrobial Pharmacodynamics and Therapeutics group, Department of Pharmacology and Therapeutics, University of Liverpool, Liverpool, UK; Mucosal and Vascular Immunology Group, Malawi Liverpool Wellcome Programme, Blantyre, Malawi

**Keywords:** amphotericin B, cryptococcal meningitis, immunomodulation, pharmacodynamics, pharmacokinetics

## Abstract

**Background:**

Host immune responses to HIV-associated cryptococcal meningitis are critical in disease outcome. Their interaction with antifungal drug exposure is poorly understood. This study explored associations between immune biomarkers, antifungal drug exposure, and clinical outcomes in HIV-associated cryptococcal meningitis.

**Methods:**

We analyzed serial plasma and cerebrospinal fluid immune biomarkers from 64 participants recruited from the AMBITION-cm trial. We estimated individual-level exposure to amphotericin B, flucytosine, and fluconazole. Associations between immune biomarkers, pharmacokinetic parameters, and clinical outcomes were evaluated.

**Results:**

An inflammatory cerebrospinal fluid response, characterized by coordination between tumor necrosis factor-α, granulocyte colony-stimulating factor, and interleukin-7 signaling, was linked to low fungal burden, low intracranial pressure, and survival. However, the value of specific immune biomarkers did not predict EFA or mortality. Exposure to amphotericin B was significantly associated with EFA.

**Conclusions:**

Favorable clinical outcomes from HIV-associated cryptococcal meningitis are associated with coordinated inflammatory and cytotoxic responses in the central nervous system. Antifungal drug exposure was the dominant predictor of EFA.

All-cause mortality from HIV-associated cryptococcal meningitis remains in the region of 25% to 36% at 10 weeks [1, 2]. The host immune response is central to the pathogenesis of cryptococcosis and determines whether infection is contained within pulmonary granulomas or becomes disseminated, including to the central nervous system (CNS) [3]. In advanced HIV, dysregulation of CD4+ T cells interferes with inflammatory signaling, enabling evasion of immune surveillance, persistence of infection, and CNS invasion [4, 5]. T-helper (Th) 1 inflammatory responses, including release of proinflammatory cytokines such as interferon-gamma (IFN-γ), tumor necrosis factor-alpha (TNF-α), interleukins (ILs)-2, 6, 8, and 17, are associated with enhanced early fungicidal activity (EFA) and reduced mortality [6–8]. This signalling promotes classical M1 macrophage polarization with attendant phagocytosis and fungicide [9, 10]. In mice, a predominance of CD4+ cells producing Th2-type responses with anti-inflammatory signalling and alternative (M2) macrophage activation is associated with detrimental outcomes [11, 12]—although this has not been consistently replicated in humans. Deficiency of Th1-type cytokines is associated with increased mortality in patients with cryptococcal meningitis [6, 13].

The relationships between T-cell responses, macrophage activation pathways, and clinical manifestations from cryptococcal infection are not straightforward. Additional complexity is added in the setting of antifungal therapy, which reduces fungal burden and impacts the associated immune response [14, 15]. In this study, we aimed to provide insight into the relative influence of the host response and antifungal drug exposure on clinical outcomes from HIV-associated cryptococcal meningitis. Immune biomarkers were measured in plasma and cerebrospinal fluid (CSF) collected serially from participants during the first 14 days of treatment. Posterior estimates of individual-level exposure to fluconazole, flucytosine, and amphotericin B were derived from population pharmacokinetic (PK) models. We examined the associations between immune biomarkers, antifungal drug exposure, and clinical outcomes from cryptococcal meningitis.

## MATERIALS AND METHODS

### Study Participants and Clinical Procedures

Participants were recruited from Queen Elizabeth Central Hospital in Blantyre, Malawi. People living with HIV with a confirmed first episode of cryptococcal meningitis were recruited as part of the phase III AMBITION-cm trial [1]. Participants were randomized 1:1 to receive induction therapy with either a single dose of liposomal amphotericin B (Ambisome, Gilead Sciences; 10 mg/kg/day) followed by 14 days of flucytosine (100 mg/kg/day) plus fluconazole (1200 mg/day)—the intervention arm—or 7 days of amphotericin B deoxycholate (1 mg/kg/day) plus flucytosine (100 mg/kg/day), followed by 7 days of fluconazole (1200 mg/day)—the control arm.

### Patient Consent Statement

All patients who had capacity provided written, informed consent for participation in the AMBITION-cm trial. If patients were incapacitated, consent was obtained from a next of kin with legal responsibility and then participants were reconsented if possible according to their clinical status. Ethical approval for AMBITION-cm was obtained from the Malawi National Health Sciences Research Committee (#1907) and the Research Ethics Committee of the London School of Hygiene and Tropical Medicine (#14355).

### Collection and Measurement of Soluble Biomarkers

Blood samples for measurement of immune biomarkers were collected on day 1 (before initiation of study drugs), day 7, and day 14. Blood samples for PK were collected on days 1 and 7 of the study at 0, 2, 4, 7, 12, and 23 hours after drug administration. CSF was obtained by lumbar puncture (LP) for measurement of immune biomarkers and PK before initiation of study drugs (day 1) and on days 7 and 14. Additional LPs were performed if clinically indicated for management of raised intracranial pressure. CSF obtained from those LPs was also analyzed.

Plasma and CSF samples were processed within 1 hour of collection. Samples were centrifuged at 3000 rpm (1500*g*) for 10 minutes at 4 °C before freezing at −80 °C until analysis on site at the Malawi Liverpool Wellcome Programme. We used the Luminex multianalyte platform (Luminex, Merck Millipore, Herts, UK) to measure IFN-γ, TNF-α, granulocyte-macrophage colony-stimulating factor (GM-CSF), granulocyte colony-stimulating factor (G-CSF), IL-2, IL-4, IL-5, IL-6, IL-7, IL-8 (CXCL8), IL-10, IL-15, IL-17A, IL-12p40, IL-12p70, IL-1 receptor antagonist (IL-1ra), IFN-γ inducible protein 10 (IP-10/CXCL10), vascular endothelial growth factor (VEGF)-A, monocyte chemoattractant protein 1/chemokine (C-C motif) ligand 2 (MCP-1/CCL2), macrophage inflammatory protein (MIP)-1a/CCL3, MIP-1b/CCL4, and RANTES/CCL5, according to the manufacturer's instructions.

Quantitative cryptococcal culture was performed on days 1, 7, and 14 using serial dilutions of 100 μL CSF with colony counting after 48 hours of growth at 30 °C. For this substudy, participants were followed up for 10 weeks with respect to mortality outcomes.

### Pharmacokinetic Analyses

The sampling, bioanalytical, and modeling techniques used for this study are explained in detail elsewhere [16–18]. In brief, concentration-time data for fluconazole, flucytosine, and amphotericin B in plasma (all drugs) and CSF (fluconazole and flucytosine only) were modeled using the nonparametric adaptive grid algorithm of the program Pmetrics [19] version 2.0.2 for R. Drug exposure was estimated by calculating the area under the concentration-time curve (AUC) using the Bayesian posterior PK predictions for each participant to perform trapezoidal approximation in Pmetrics. These same estimates of drug exposure were used by our group in another recent publication [18].

### Data Analysis

Two statistical techniques were employed to reduce the dimensionality of the large set of soluble biomarkers: principal component analysis (PCA) and network analysis. PCA derives linear functions (principal components [PCs]) that capture the variance in a dataset and that are unrelated to one another, increasing the interpretability of the data while minimizing information loss and avoiding multiple comparisons [20]. The purpose of performing PCA in this case was to reduce the “noise” created by relatively uninformative variables and to collapse variables that were correlated to one another, enabling simpler models of association between informative immune biomarkers, pharmacokinetic variables, and clinical outcomes. Network analysis places significance on the associations between variables, highlighting clusters of variables that are more closely related to one another than to other variables. The purpose of the network analyses performed here was to identify coordinated immune responses associated with clinical outcomes. The network analyses presented here included only those cytokines that were strongly correlated (Pearson's correlation coefficient, *r*, ≥0.70; *P* < .05).

Prior to PCA and network analysis, analyte concentrations were log_10_ transformed and normalized to the mean value for that analyte across all timepoints. A “slope” value was calculated for each biomarker for each participant as a linear regression of the log_10_ transformed, normalized concentration of that biomarker over time. PCA and network analyses were performed on biomarker values from day 1 of the study and on the slope values.

Several clinical outcomes served as dependent variables: fungal burden at baseline prior to study drug administration, opening intracranial pressure at baseline, EFA (calculated as a linear regression of log_10_ CFU/mL of CSF over the first 14 days of therapy) and mortality (followed up to 10 weeks). Regression models were used to examine the relationship between PCs, posterior PK predictions, and clinical outcome variables. Associations with opening pressure were adjusted for baseline fungal burden in CSF. Associations with EFA were adjusted for both baseline fungal burden and study arm. Associations with time to death were examined using Cox proportional hazards models and adjusted for baseline fungal burden because this is consistently associated with mortality from HIV-associated cryptococcal meningitis [21]. Interaction terms were included between each immune response PC and baseline fungal burden, to account for the presumed impact of the former on the latter. Statistical significance was defined as *P* < .05. Original data are available online within the Supplementary material.

## RESULTS

### Participants

Between November 2018 and October 2019, 64 participants were recruited to this substudy of the AMBITION-cm trial—31 participants in the control arm and 33 in the single-dose liposomal amphotericin arm. The median age was 36 years (interquartile range [IQR] 33–41 years) and 37% of participants were female. Median weight was 50 kg (IQR 47–56 kg). Median CD4 cell count was 39 cells/mm^3^ (IQR 21–83 cells/mm^3^, n = 57). At the time of recruitment, 35 participants were taking antiretroviral treatment (ART) and 29 were not taking ART. The cumulative case fatality rate was 9% at 2 weeks (6/64) and 28% at 10 weeks (18/64).

### Plasma and CSF Immune Biomarkers

**Figure 1.**
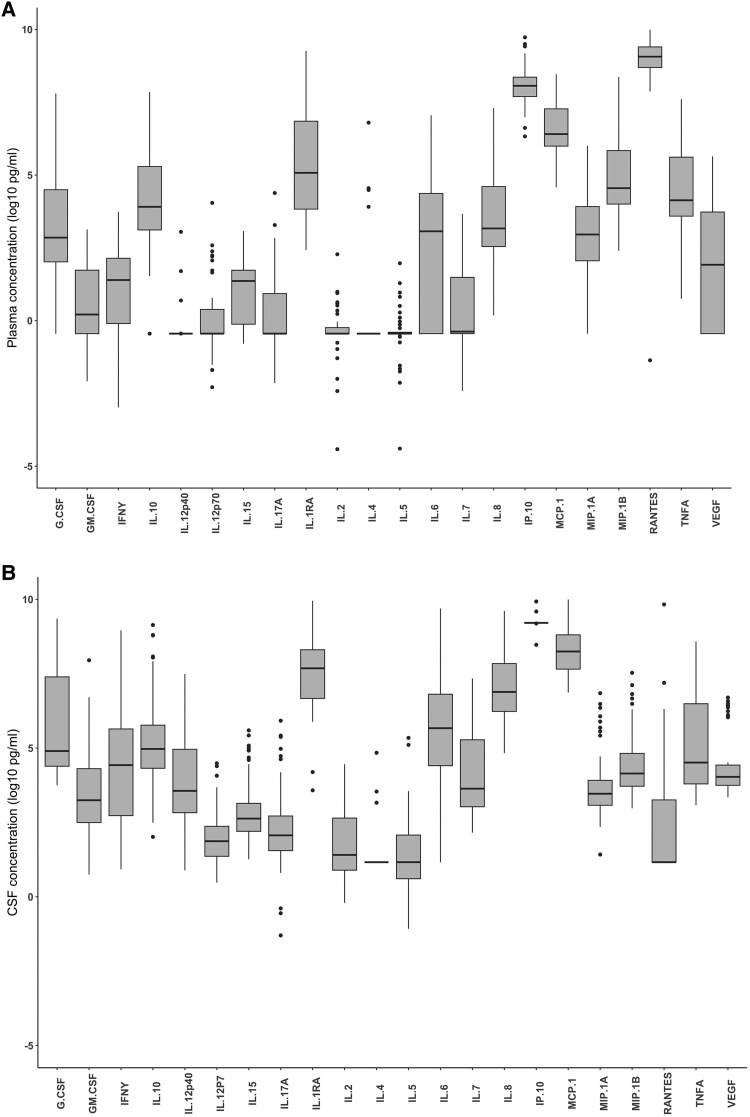
Baseline immune biomarker concentrations. Baseline soluble biomarker concentrations in *A*, plasma and *B*, CSF in patients with HIV-associated cryptococcal meningitis. Boxes indicate median and interquartile range. Whiskers represent 10th and 90th percentiles. Abbreviation: CSF, cerebrospinal fluid.

**Figure 2.**
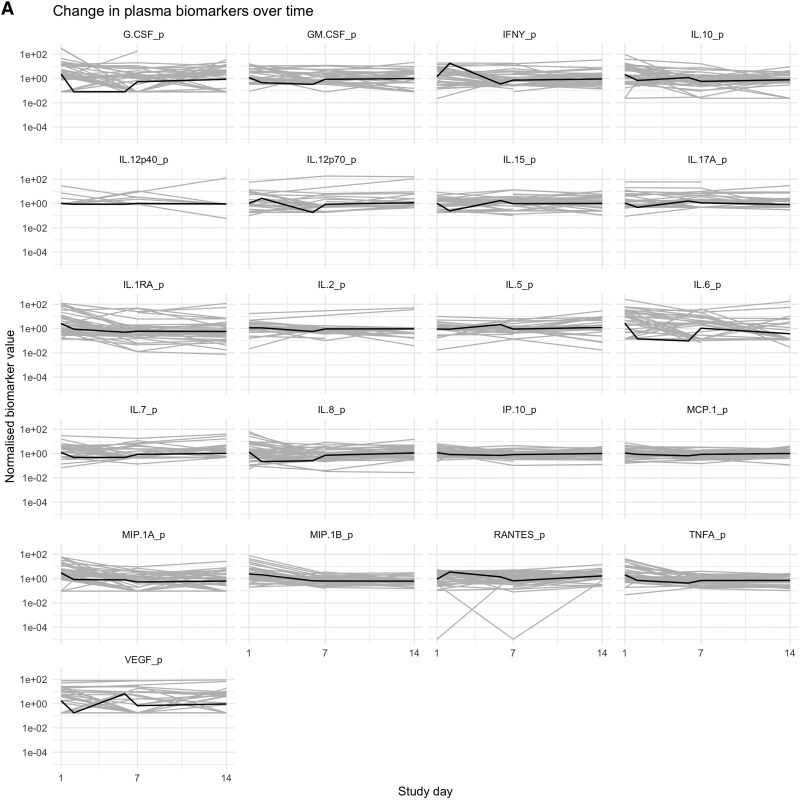

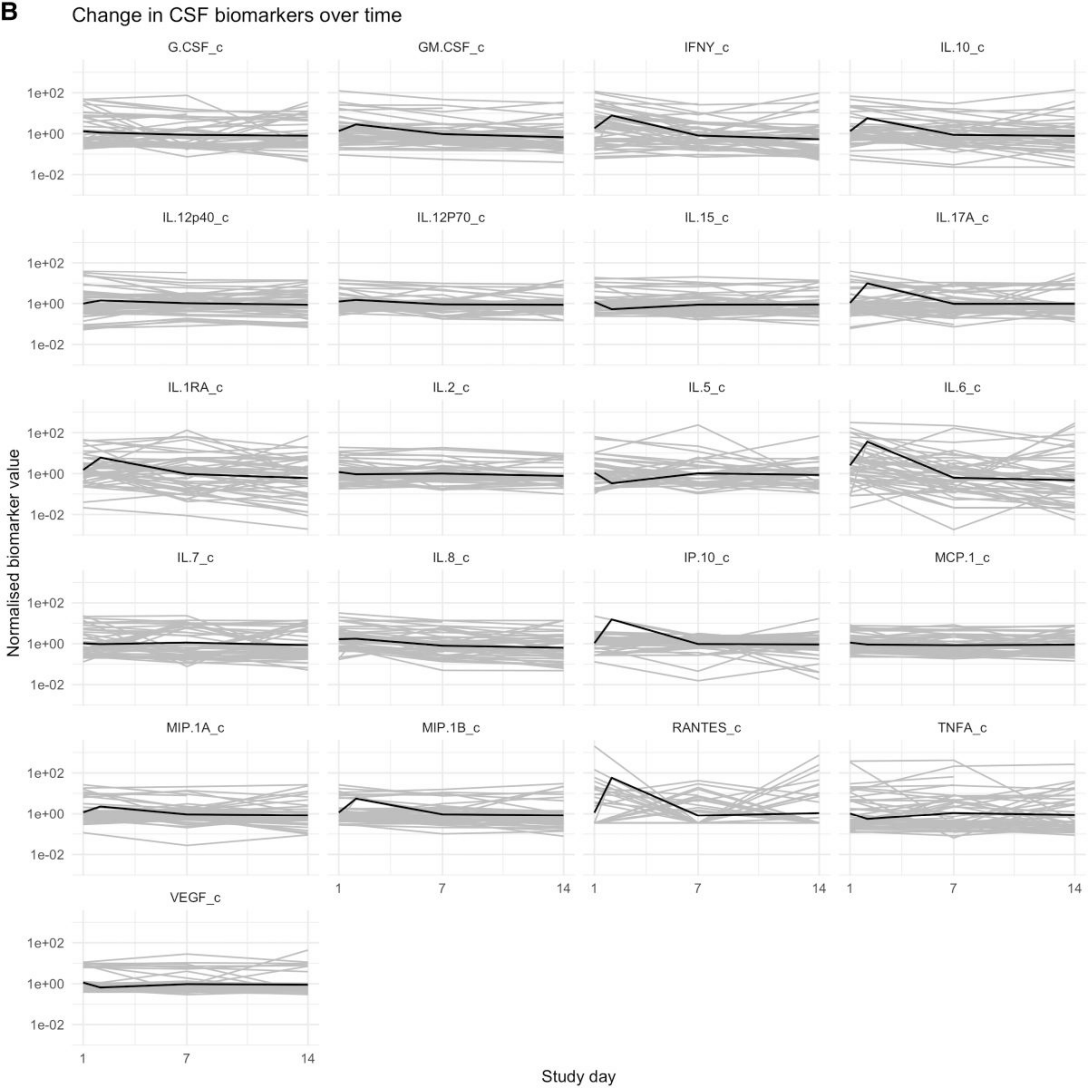
Change in biomarker values over time in *A*) plasma and *B*) cerebrospinal fluid. Gray lines represent individual patient biomarker values over time; black line is the mean across the patient population.

The range of biomarker concentrations at baseline (day 1) in plasma and CSF are shown in Figure 1. The change in biomarker values over time is depicted in Figure 2.

### Posterior Estimates of Drug Exposure

The final PK models revealed substantial interindividual variability in drug exposure in this critically unwell population. The median amphotericin B AUC_0–24_ in plasma was 0.028 g/L/h (IQR 0.014–0.540); flucytosine median AUC_144-168_ in CSF was 0.579 (IQR 0.343–0.813) and fluconazole median AUC_144-168_ was 5.504 (IQR 3.902–6.752). These time windows were chosen to capture the high peak plasma concentration of amphotericin B in the first 24 hours for those participants in the intervention arm and to capture steady state of flucytosine and fluconazole in the CNS.

### Principal Component Analysis

#### Baseline Biomarker Values in Plasma and CSF

**Figure 3.**
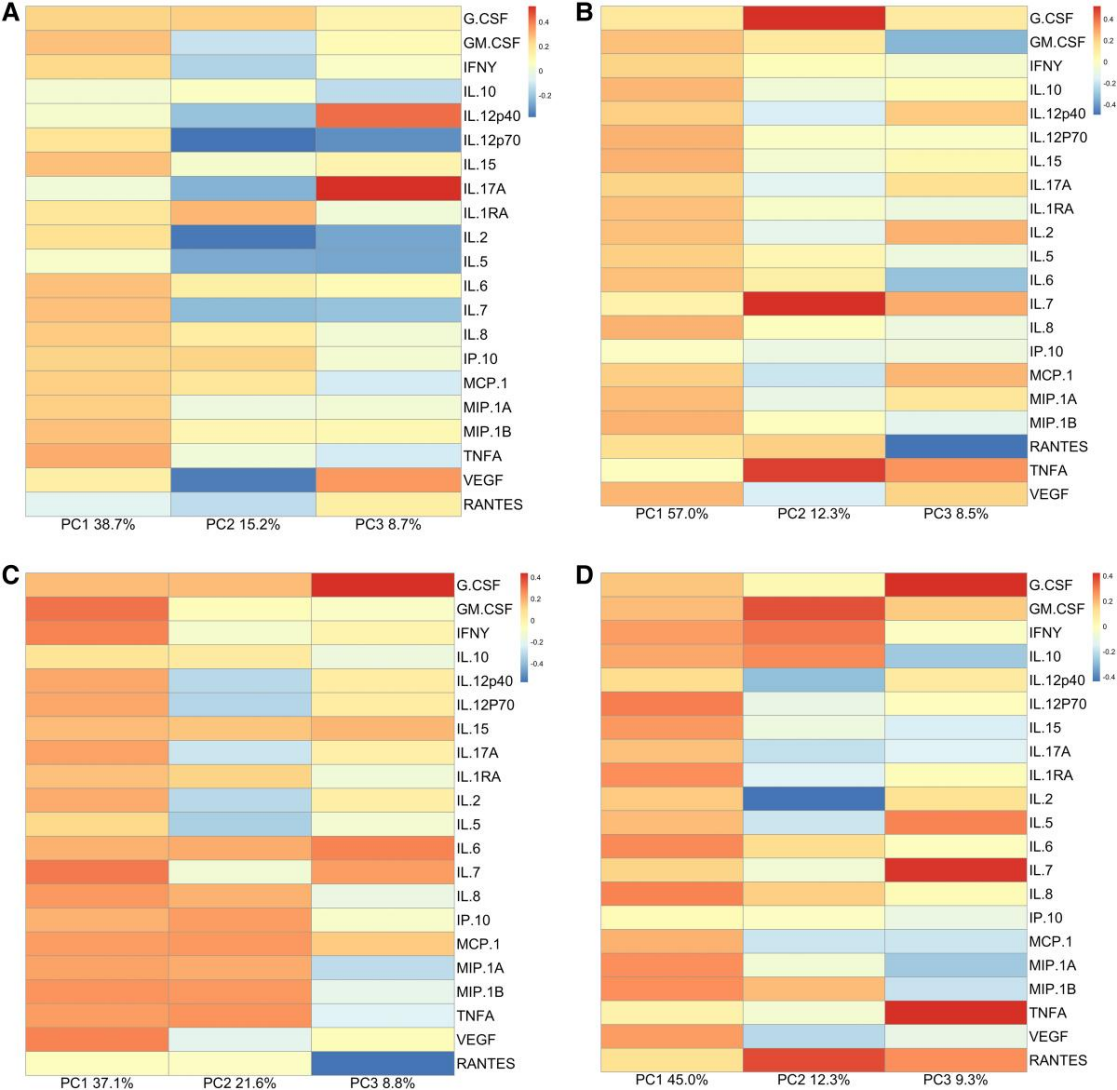
Principal component analysis. *A*, Plasma, day 1: PCs 1, 2, and 3 accounted for 38.7%, 15.2%, and 8.7% of the variance, respectively. The majority of soluble biomarkers in the dataset contribute relatively homogeneously to the variance in PC1. PC2 shows strongly negative loading scores in IL-12p70, IL-2, and VEGF, with PC3 predominated by a strongly positive loading score in IL-17A. *B*, CSF, day 1: PCs 1, 2, and 3 accounted for 57.0%, 12.3%, and 8.5% of the variance, respectively. Again, there is relatively homogeneous contribution to variance in PC1. In PC2 is overwhelmingly characterized by positive loadings in G-CSF, IL-7, and TNF-α. PC3, in contrast, is predominantly defined by a negative loading score in RANTES, as well as a moderately positive score in TNF-α. *C*, Plasma, slope values: PCs 1, 2, and 3 accounted for 37.1%, 21.6%, and 8.8% of the variance, respectively. PC1 was again characterized by relatively homogeneous, moderately positive loading scores across the majority of biomarkers. PC2 comprised negative loading scores in IL-2, IL-5, IL-12p40, and IL-12p70. PC3 was characterized by strong positive loading scores in the slope values for IL-6 and IL-7, and negative scores in the slope values for IP-10 and RANTES. *D*, CSF, slope values: PCs 1, 2, and 3 accounted for 45.0%, 12.3%, and 9.3% of the variance, respectively. PC1 was characterized by moderately positive loading scores across the majority of biomarkers. PC2 was characterized by relatively strong contributions from GM-CSF and RANTES, with a strong negative score for IL-2. PC3 comprised strong positive loading scores for G-CSF, IL-7, and TNF-α. Abbreviations: GM-CSF, granulocyte-macrophage colony-stimulating factor; IL, interleukin; PC, principal component; TNF, tumor necrosis factor; VEGF, vascular endothelial growth factor.

PCA of plasma biomarkers from study day 1 demonstrated that 62.6% of the overall variance in the dataset was accounted for by a combination of PC1 (38.7%), PC2 (15.2%), and PC3 (8.7%). PC1 comprised a large group of cytokines that contributed relatively moderate, positive loading scores, implying that all variables contributed similar information to that PC. The variance in PC2 was primarily accounted for by negative loading scores for IL-12p70, IL-2, and VEGF, indicating that the absence of those variables contributed to PC2. PC3 was overwhelmingly described by a strong contribution from IL-17A, with moderate loading scores in other biomarkers (Figure 3*A*).

In CSF, 77.8% of the variance in the data from day 1 was accounted for by the combination of PC1 (57.0%), PC2 (12.3%), and PC3 (8.5%) (Figure 3*B*). A large group of biomarkers of Th1-, Th2-, and Th17-type responses drove the variance in PC1; the contribution of each of those biomarkers to PC1 was primarily positive and relatively homogeneous. The variance in PC2, in contrast, was chiefly accounted for by positive loading scores in a group of just 3 biomarkers: G-CSF, IL-7, and TNF-α. The chemokine RANTES was strongly negatively correlated with PC3.

#### Dynamic Biomarker Values in Plasma and CSF

Plasma biomarker concentrations were available for 39 participants on day 1, 41 on day 7, and 37 on day 14. In CSF, biomarker concentrations were available from 61 participants on day 1, 55 patients on day 7, and 53 participants on day 14.

In plasma, the first 3 PCs accounted for 67.5% of the variance in the dataset describing change in immune biomarkers over time: PC1 37.1%, PC2 21.6%, and PC3 8.8% (Figure 3*C*). In CSF, 66.6% of the total variance was accounted for by the first 3 PCs: PC1 45.0%, PC2 12.3%, and PC3 9.3% (Figure 3*D*). In both plasma and CSF, PC1 was again characterized by relatively homogeneous, moderately positive loading scores across all biomarkers, implying that all variables contributed similar information to that PC. In plasma, PC2 comprised negative loading scores for IL-2, IL-5, IL-12p40, IL-12p70, and IL-17A, indicating that the absence of those variables contributed to PC2. PC3 in plasma was dominated by a strong positive loading score for G-CSF (strong contribution to PC3) and a negative score in RANTES (absence of RANTES contributed to PC3). PC2 in the slope dataset from CSF values was characterized by relatively strong contributions from GM-CSF and RANTES, with a strong negative score for IL-2. PC3 comprised strong positive loading scores for G-CSF, IL-7, and TNF-α—the same biomarkers that presented the highest loading scores in the PCA of day 1 biomarkers in CSF.

### Network Analysis

#### Baseline Biomarkers

**Figure 4.**
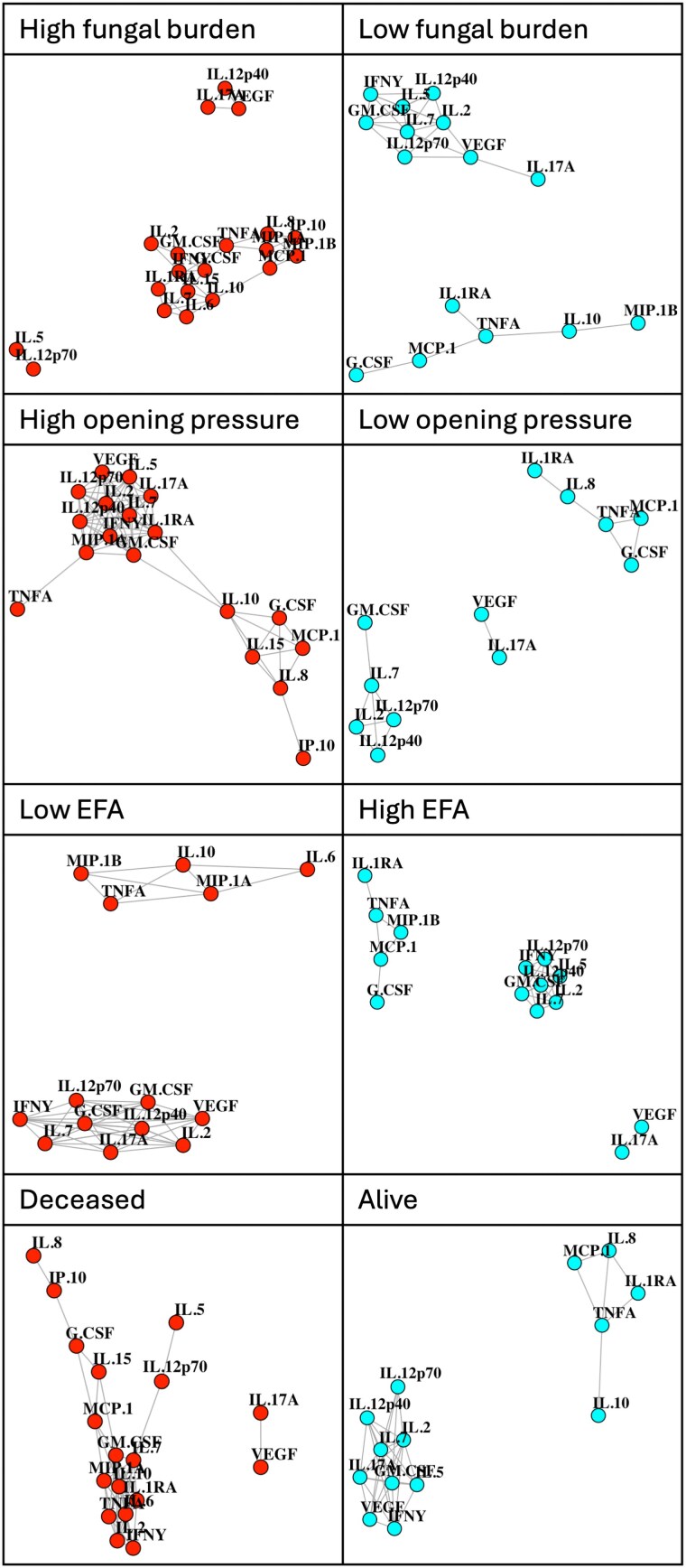
Network analyses showing the associations among immune biomarkers in plasma at baseline. Network plots showing associations between baseline levels of immune biomarkers in plasma in patients categorized according to clinical outcomes. Patients with baseline fungal burden greater than or equal to 6 log_10_ colony forming units (CFU) per mL cerebrospinal fluid (CSF); patients with baseline fungal burden less than 6 log_10_ CFU per mL CSF; patients with lumbar opening pressure greater than or equal to 30 cm H_2_O; patients with lumbar opening pressure less than 30 cm H_2_O; patients with early fungicidal activity (EFA) slower than −0.2 log_10_ CFU/mL/day; patients with EFA faster than or equal to −0.2 log_10_ CFU/mL/day; patients who died by 10 wk into the study; patients who survived to 10 wk. Color scheme is purely for presentation.

Network analysis of baseline plasma biomarkers suggested a particular pattern of biomarkers in participants with high fungal burden and those who died before 10 weeks of follow-up (Figure 4). In these participants, there were 3 distinct clusters of immune biomarkers: (1) IL-17A, IL-12p40 and VEGF; (2) IL-5 and IL-12p70; and (3) all remaining biomarkers. Of note, the first of these distinct clusters (IL-17A, IL-12p40, and VEGF) was also seen in patients with high EFA, suggesting that this clustering alone was not consistently related with either favorable or poor clinical outcomes. IL-17A, IL-12p40, and VEGF also clustered together in participants who were not taking ART, but not in those who were taking ART (Supplementary Figure 1). These are the same 3 biomarkers that contributed positive loading scores to PC3 in the PCA of baseline biomarker values in plasma.

**Figure 5.**
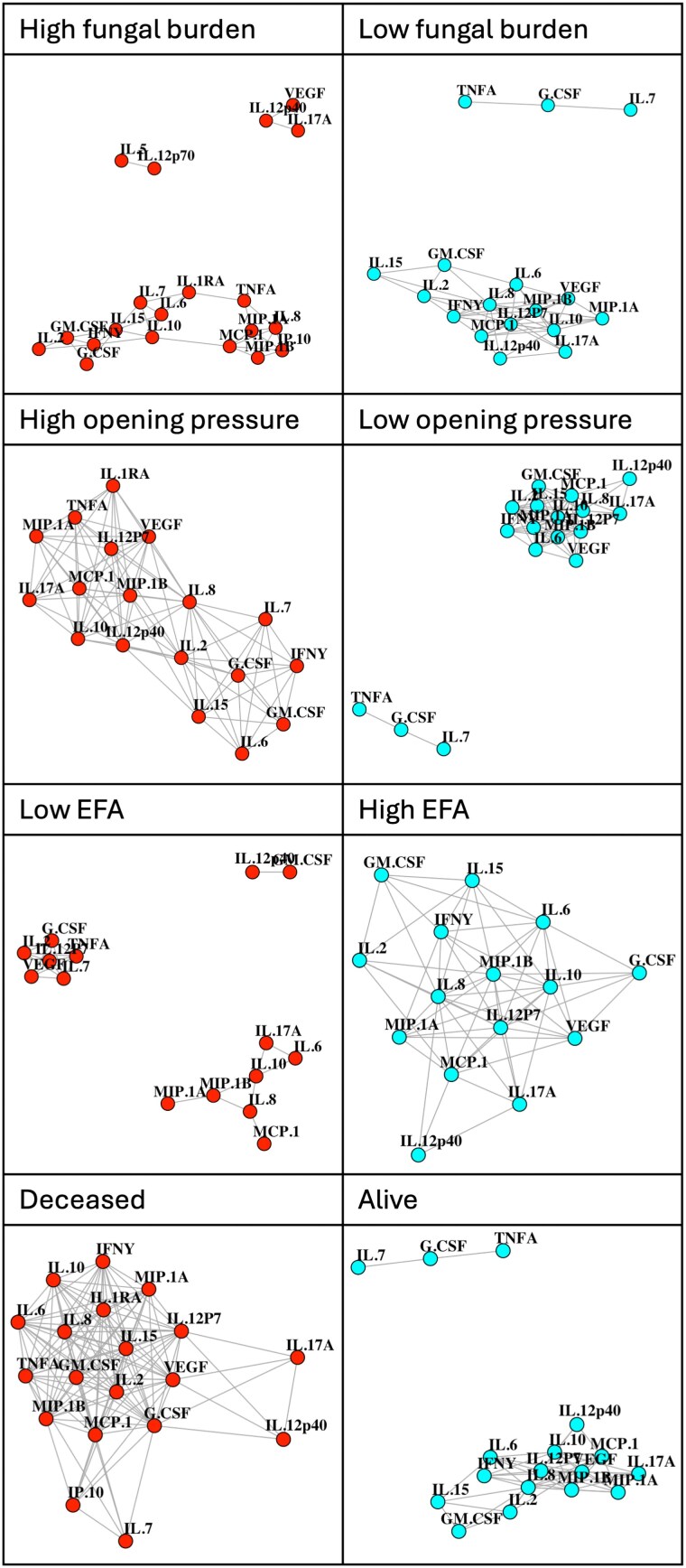
Network analyses showing the associations among immune biomarkers in cerebrospinal fluid (CSF) at baseline. Network plots showing associations between baseline levels of immune biomarkers in CSF in patients categorized according to clinical outcomes. Patients with baseline fungal burden greater than or equal to 6 log_10_ colony forming units (CFU) per mL CSF; patients with baseline fungal burden less than 6 log_10_ CFU per mL CSF; patients with lumbar opening pressure greater than or equal to 30 cm H_2_O; patients with lumbar opening pressure less than 30 cm H_2_O; patients with early fungicidal activity (EFA) slower than −0.2 log_10_ CFU/mL/day; patients with EFA faster than or equal to −0.2 log_10_ CFU/mL/day; patients who died by 10 wk into the study; patients who survived to 10 wk. Colour scheme is purely for presentation.

Network analysis of day 1 CSF immune biomarker values revealed a more consistent pattern (Figure 5). In participants with favorable clinical outcomes (low baseline fungal burden, low opening pressure, fast EFA, and survival at 10 weeks) G-CSF, IL-7, and TNF-α clustered together, distinct from other biomarkers, indicating that these cytokines were more closely correlated to one another than they were to other biomarkers. This pattern was not present in participants with high baseline fungal burden, high CSF opening pressure, or in those who had died within 10 weeks of study enrolment. However, the same pattern was seen in participants with low EFA, suggesting that this clustering did not distinguish between patients with slow versus fast pharmacodynamic responses. In addition, the pattern was present regardless of ART status. G-CSF, IL-7, and TNF-α are the same biomarkers that contributed strongly to PC2 in CSF.

### Network Analysis

#### Dynamic Biomarker

Values Network analysis of the slope of each biomarker value revealed less consistent patterns than the networks of baseline biomarkers, in terms of associations with favorable versus poor clinical outcomes (Supplementary Figure 2). Overall, there was not a convincing trend in these analyses.

In CSF, the pattern of G-CSF, IL-7, and TNF-α clustering together, distinct from other biomarkers, was seen in biomarker slope values (in keeping with the baseline dataset) in patients who survived to 10 weeks. This pattern was not observed in patients who were deceased at 10 weeks. In keeping with the baseline dataset, this clustering did not distinguish between patients with slow versus fast EFA (Supplementary Figure 3). There was no difference in the slope values of G-CSF, IL-7, or TNF-α between patients in the control versus single-dose arm of the study (data not shown).

### Relationships Between Principal Components, Pharmacokinetic Variables and Clinical Outcomes

In regression models, we used the PCs from baseline data rather than the PCAs of slope values, since the PCA of baseline data identified the most consistent patterns.

There was a statistically significant association between PC3 in plasma and baseline fungal burden and between PC2 in CSF and baseline fungal burden (Table 1). Neither baseline fungal burden nor any of the PCs from plasma or CSF data were associated with lumbar opening pressure in univariate regression (data not shown) or multivariate regression (Supplementary Table 1).

**Table 1.** Adjusted Multivariable Regression Model Examining Predictors of Fungal Burden at Baseline

	Regression Coefficient	Standard Error	*P* Value
Plasma PC1	0.25	0.14	.08
Plasma PC2	0.12	0.16	.46
Plasma PC3	**0.44**	**0**.**21**	.**04**
CSF PC1	−0.15	0.10	.15
CSF PC2	**−0**.**35**	**0**.**17**	.**04**
CSF PC3	0.21	0.22	.34

Bold values indicate statistically significant associations. Fungal burden measured in log_10_ CFU/mL.

Abbreviations: CFU, colony forming unit; CSF, colony-stimulating factor; PC, principal component.

Adjusting for baseline fungal burden and exposure to each antifungal drug, none of the immune data PCs was associated with EFA. However, both baseline fungal burden and exposure to amphotericin B were significantly associated with increased EFA: for each log_10_ CFU/mL increase in fungal burden, EFA increased by 0.07 log_10_ CFU/mL/day (standard error 0.01, *P* < .001); for each g/L/h increase in amphotericin B AUC_0-24_, EFA increased by 0.14 log_10_ CFU/mL/day (standard error 0.04, *P =* .001); Table 2.

**Table 2.** Adjusted Multivariable Regression Model Examining Predictors of Early Fungicidal Activity

	Regression Coefficient	Standard Error	*P* Value
Baseline fungal burden (log_10_ CFU/mL)	**−0**.**07**	**0**.**01**	**1.65 E^−5^**
Study arm: single dose AmBisome	Odds ratio 1.03	0.08	.71
Plasma PC1	0.00	0.01	.93
Plasma PC2	−0.01	0.01	.67
Plasma PC3	−0.02	0.02	.19
CSF PC1	−0.01	0.01	.33
CSF PC2	0.00	0.01	.83
CSF PC3	−0.01	0.01	.61
Amphotericin AUC_0-24_ (g.L/h)	**−0**.**14**	**0**.**04**	.**04**
Flucytosine AUC_144-168_ (g.L/h)	−0.04	0.02	.11
Fluconazole AUC_144-168_ (g.L/h)	0.01	0.01	.29

Bold values indicate statistically significant associations. EFA measured in log_10_ CFU/mL/day.

Abbreviations: AUC, area under the concentration-time curve; CFU, colony forming unit; PC, principal component.

A Cox proportional hazard model did not identify any statistically significant independent predictors of survival time among the PK parameters or PCs in univariate or multivariate analysis (data not shown).

## DISCUSSION

We present a comprehensive analysis of the immunophenotype both in the peripheral circulation and in the CNS of patients with HIV-associated cryptococcal meningitis. There was a notable lack of dynamism in immune biomarker values over the first 14 days of treatment in either plasma or CSF, and analysis of the slope values of each biomarker did not yield significant information above that provided by analysis of baseline values. An explanation for this may be that the immune response to infection with *Cryptococcus* spp. is shaped prior to diagnosis and the initiation of antifungal therapy, with fluctuations in responses relatively minor once disease is established.

In plasma, IL-12 p40, IL-17A, and VEGF contributed strongly to the PC that was statistically significantly associated with high baseline fungal burden—an established predictor of mortality in cryptococcal meningitis [18, 21, 22]. This was also reflected in the network analysis of plasma biomarkers at baseline. This combination of cytokines suggests Th1 and Th17 T-cell differentiation, neutrophil, and monocyte recruitment, activation of glial cells, and amplification of inflammatory signalling. Indeed, IL-12 is an essential cytokine for protective Th1 responses in murine models of cryptococcosis [23]. VEGF can additionally disrupt the blood–brain barrier to enable inflammatory cells and biomarkers to infiltrate the CNS. In contrast with other investigators [6], our data did not convincingly associate this pro-inflammatory signature in plasma with clinical benefit. This may be because the immune response in plasma does not reflect activity at the primary site of pathology in the CNS. Alternatively, it may be a function of the small number of participants in our cohort relative to the number of measured biomarkers.

In keeping with other cohorts, favorable clinical outcomes in our study were associated with a coordinated inflammatory and cytotoxic response in the CNS [6, 24]. TNF-α is a core Th1-type inflammatory and cytotoxic mediator, involved in protection against a range of intra- and extracellular pathogens [25–29]. TNF-α stimulates IFN-γ production, the beneficial role of which is well documented in patients with HIV/CM [7, 30]. G-CSF promotes the differentiation of stem cells into cytotoxic neutrophils and monocytes [31, 32]. In vitro experiments suggest that G-CSF may also have direct immunomodulatory properties: the addition of G-CSF to neutrophils, monocytes, or monocyte-derived macrophages enhances cryptococcal killing both in the presence and absence of azole antifungal treatment [33]. IL-7 is essential for lymphopoiesis and plays a key role in homeostasis of T cells and inflammatory signalling networks [34–36]. There is evidence to suggest that amphotericin B has immunomodulatory effects and that these effects differ between amphotericin B formulations [14]. We did not design this study to detect those differences and indeed did not observe differences in these key inflammatory cytokines between study arms.

The immune response to HIV-associated cryptococcal meningitis is marked by significant interindividual heterogeneity. Identifying safe, generalizable targets for immunomodulatory therapy is hugely challenging. A randomized controlled trial of adjunctive IFN-γ alongside amphotericin B deoxycholate and flucytosine demonstrated improved EFA and a trend toward reduced mortality in the IFN-γ groups [7]. However, this intervention is not in routine clinical use, in large part because of a lack of robust evidence of mortality benefit. Larger trials of adjunctive IFN-γ alongside a backbone of gold-standard antifungal treatment, including mortality endpoints, are required to explore this further. More data are also required to identify those who are most likely to benefit from adjunctive IFN-γ. It is not clear, for example, whether ART status would alter the response to immunomodulation.

From the point of diagnosis of cryptococcal meningitis, the key intervention to improve outcomes remains the administration of potent antifungal therapy. Our analyses in CSF suggest a consistent immune signature that distinguishes between patients with high- and low-baseline fungal burden, high and low lumbar opening pressure, and mortality but fails to distinguish between high and low EFA. A plausible explanation for this is that antifungal drug exposure is more predictive of EFA than the immune response in the CNS. This hypothesis is supported by our multivariable analysis, with amphotericin B exposure remaining a significant predictor of EFA when adjusting for baseline fungal burden, immune signatures, and exposure to fluconazole and flucytosine. We recently examined the relative contribution of pathogen genomics and antifungal pharmacokinetics to EFA in patients with HIV-associated cryptococcal meningitis and similarly concluded that the most significant contributor is exposure to amphotericin B [18].

This study has limitations. First, our cohort was relatively small in comparison to the number of clinical variables collected. We mitigated the statistical impact of this to a degree by implementing PCA and network analyses before undertaking regression analyses. Second, our estimates of amphotericin B exposure in CSF were derived from plasma samples. This is because measured levels of amphotericin B are negligible in CSF and the PK profile obtained from those samples is thought unlikely to reflect exposure in brain parenchyma [37, 38]. Our practice is therefore to measure in plasma and model CNS exposure. Third, we were unable to account for microbiological features such as strain lineage and phenotype in our analysis because our sample size was insufficient to support the addition of further datapoints. These data are published elsewhere [18].

In summary, our analysis supports an association between an inflammatory, Th-1 type immune response in the CSF and favorable clinical outcomes from HIV-associated cryptococcal meningitis. Our data emphasize the complexity of the immune response to HIV-associated cryptococcal meningitis and provide insight into the challenges of designing host-directed therapies. Furthermore, our data suggest that the impact of exposure to amphotericin B overshadows the impact of immune signatures either peripherally, or at the site of infection, on EFA. In the design of future treatment protocols for HIV-associated cryptococcal meningitis, optimized antifungal drug exposure should be an absolute priority. Access to potent anticryptococcal drugs is essential for all people who suffer with this disease.

## Supplementary Material

ofaf190_Supplementary_Data

## Supplementary Data

Supplementary materials are available at *Open Forum Infectious Diseases* online. Consisting of data provided by the authors to benefit the reader, the posted materials are not copyedited and are the sole responsibility of the authors, so questions or comments should be addressed to the corresponding author.
