# Supplementary material for: Relative Contribution of Pharmacokinetics and Immune Signatures to Clinical Outcomes in Patients With HIV-associated Cryptococcal Meningitis
Source: Open Forum Infect Dis. 2025 Apr 2;12(4):ofaf190. doi: 10.1093/ofid/ofaf190 (PMC12015468; doi:10.1093/ofid/ofaf190)

# Supplementary material

### Supplementary table 1: Adjusted multivariable regression model examining predictors of opening pressure at baseline.

|  | Regression coefficient | Standard error | P-value |
| --- | --- | --- | --- |
| Baseline fungal burden (log_10_ CFU/mL) | 1.13 | 1.69 | 0.51 |
| Plasma PC1 | -0.32 | 1.39 | 0.82 |
| Plasma PC2 | 1.63 | 1.59 | 0.31 |
| Plasma PC3 | -0.32 | 2.10 | 0.88 |
| CSF PC1 | 1.27 | 1.00 | 0.21 |
| CSF PC2 | -0.77 | 1.74 | 0.66 |
| CSF PC3 | 2.83 | 2.12 | 0.19 |

Opening pressure recorded as cm H_2_O.

### Supplementary figure 1: Network analyses showing the associations among immune biomarkers in plasma at baseline, according to ART status


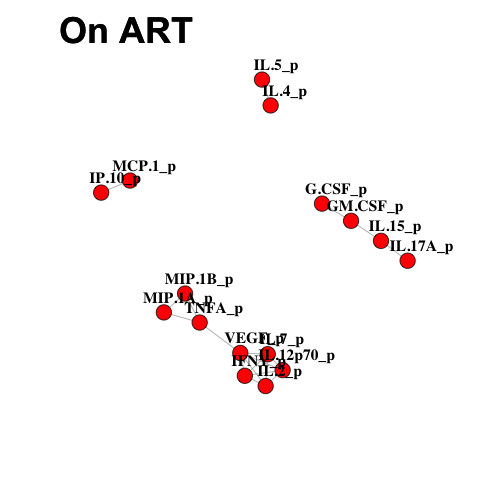


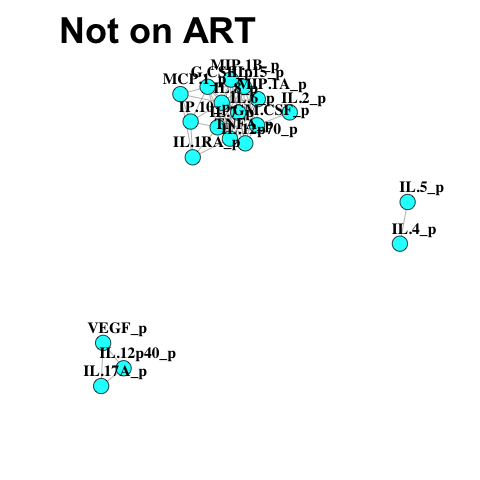


### Supplementary figure 2: Network analyses showing the associations among slopes in immune biomarkers in plasma


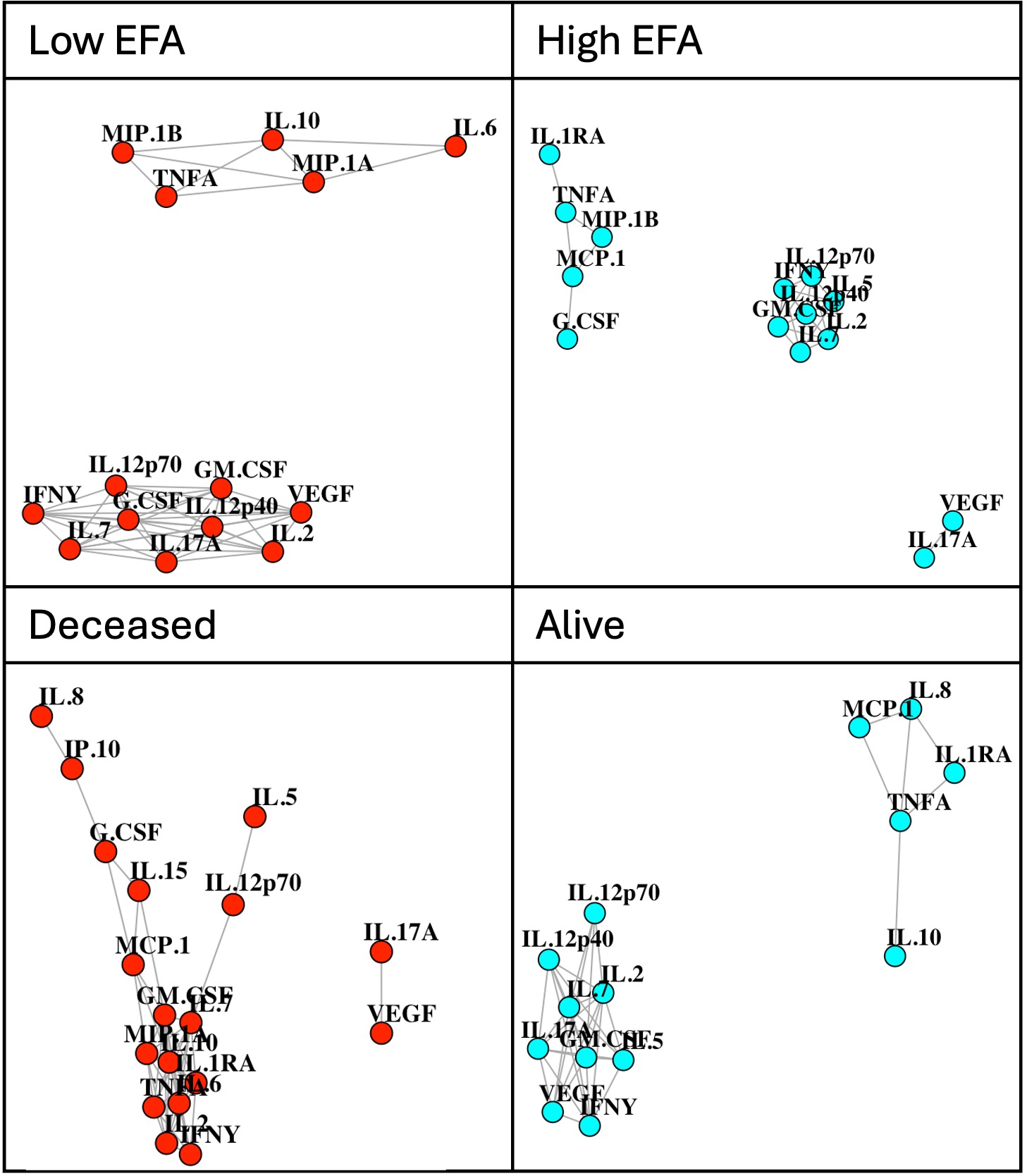


### Supplementary figure 3: Network analyses showing the associations among slopes in immune biomarkers in CSF


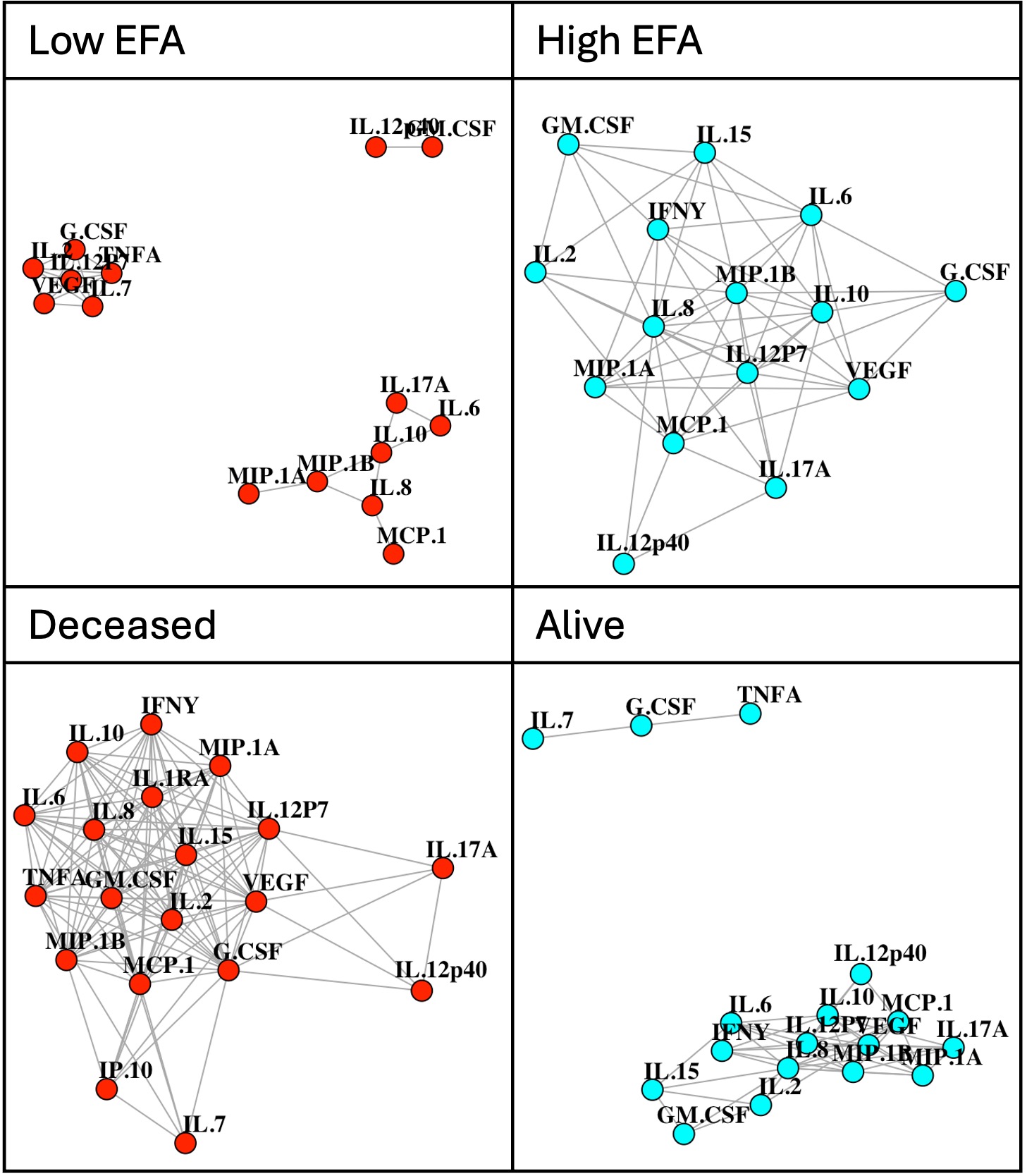

Supplement: ofaf190_Supplementary_Data [file ofaf190_supplementary_data.zip › Supplementary material.docx]
